# Role of Microstructure in Drug Release from Chitosan Amorphous Solid Dispersions

**DOI:** 10.3390/ijms232315367

**Published:** 2022-12-06

**Authors:** David Lucio, Arantza Zornoza, Maria Cristina Martínez-Ohárriz

**Affiliations:** Department of Chemistry, Faculty of Sciences, University of Navarra, Irunlarrea s/n, 31080 Pamplona, Spain

**Keywords:** amorphous solid dispersion, drug release, microstructure, diflunisal, chitosan, carboximethylchitosan

## Abstract

The unexpected dissolution behaviour of amorphous diflunisal-chitosan solid dispersions (kneading method) with respect to the crystalline co-evaporated systems is the starting point of this research. This work is an in-depth study of the diflunisal release behaviour from either chitosan or carboxymethylchitosan dispersions. The microstructure is not usually considered when designing this type of products; however, it is essential to understand the process of solvent penetration and subsequent drug release through a polymeric system, as has been evidenced in this study. In accordance with the kinetic data analysed, it is possible to conclude that the porous structure, conditioned by the sample preparation method, can be considered the main factor involved in diflunisal release. The low mean pore size (1–2 μm), low porosity, and high tortuosity of the amorphous kneaded products are responsible for the slow drug release in comparison with the crystalline coevaporated systems, which exhibit larger pore size (8–10 μm) and lower tortuosity. Nevertheless, all diflunisal-carboxymethylchitosan products show similar porous microstructure and overlapping dissolution profiles. The drug release mechanisms obtained can also be related to the porous structure. Fickian diffusion was the main mechanism involved in drug release from chitosan, whereas an important contribution of erosion was detected for carboxymethylchitosan systems, probably due to its high solubility.

## 1. Introduction

Most of the drugs currently discovered show low oral bioavailability due to poor water-solubility and belong to classes II and IV of the Biopharmaceutical Classification System (BSC) [1]. In order to resolve this drawback, the preparation of amorphous solid dispersions (ASD) using a variety of polymers has become a successful strategy to improve drug bioavailability after oral administration [2,3]. These systems not only ensure an enhancement of drug solubility but can also control the release rate. In turn, the solubility of the polymer itself is an important factor in achieving an adequate release of the active principle by maintaining supersaturation in solution, and it can condition the release mechanism. An adequate selection of the polymer is crucial to achieve an effective controlled release behaviour [4].

Several polymers commonly used in the preparation of ASDs derive from natural and abundant products such as cellulose, starch, or chitin. Chitosan (CS), a derivative from the alkaline deacetylation of chitin, is a biodegradable, biocompatible, and mucoadhesive linear polysaccharide, which is soluble in diluted acid solutions [5]. In addition, other biological properties have been reported for chitosan, such as its antitumoral and antioxidant activities [6], as well as significant antibacterial potential [7]. These properties make it a very suitable material for use in the biomedical and pharmaceutical fields [5,8]. Moreover, chitosan can be considered a porosity modulating agent during the process of drug release, creating a free volume to facilitate drug diffusion and a tailored release mechanism [9]. Chitosan is a highly versatile material, because it can be easily modified due to the amino and carboxylic groups of its backbone. In this regard, some water-soluble CS derivatives, such as carboxymethylchitosan (CMCS), have been synthesized and widely used in order to improve its performance in controlled drug delivery systems [10,11].

Diflunisal (DF) is a non-steroidal anti-inflammatory drug belonging to Class II of BCS [12], whose bioavailability is limited by its poor aqueous solubility. In addition to its extensively studied analgesic effect, recent findings have demonstrated the ability of this drug to act as an anticancer agent [13], and it is a recognised drug for the treatment of cardiac amyloidosis [14]. Diflunisal was chosen as model drug for this investigation because of its proven ability to form solid dispersions with chitosans. It has been evidenced that the drug-polymer interactions with chitosans determine the drug crystalline structure [15].

The increased drug solubility achieved through the formation of a solid dispersion is related to various kinetic and thermodynamic aspects [3]. The strategies involved include reduction of drug molecules agglomeration and particle size, removal of drug crystallinity, increased wettability, and enhancement of local drug solubility due to interactions with the polymer functional groups [16]. In addition, solid dispersions promote the maintenance of supersaturated solutions, which favour adequate drug absorption [3]. Other aspects resulting from the manufacturing process, such as porous structure, can be determinant in the dissolution behaviours of active ingredients formulated as solid dispersions. Thereby, a comprehensive study of drug release from solid dispersions would only be reached by a proper balance of all these factors, which will be involved to a different extent depending on the specific drug/carrier system and the frequently overlooked physical and microstructural properties of the solid dispersions [1]. The present study is an in-depth analysis of the factors involved in diflunisal release behaviour from DF-CS solid dispersions, considering the type of polymer employed and focusing especially on the interrelationship between the system microstructure, conditioned by the preparation method, and the dissolution behaviours. In contrast with the general assumption that amorphous dispersions will always improve the release behaviour, this paper gives value to the role of the microstructure in drug release from chitosan solid dispersions. Finally, the study was completed with an evaluation of the release mechanisms involved.

## 2. Results and Discussion

### 2.1. Diflunisal Release

**Figure 1:**
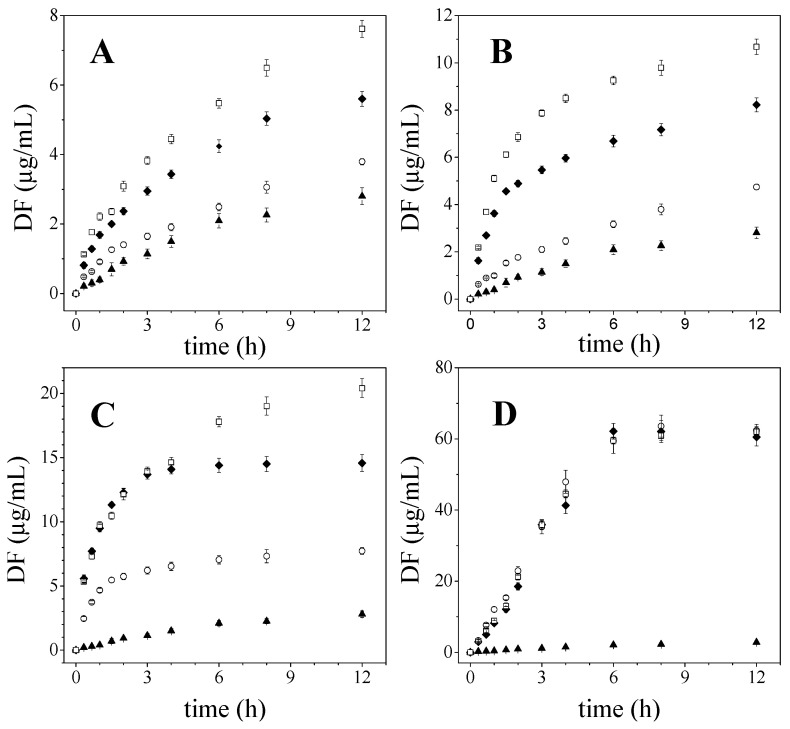
Diflunisal release profiles of: (**A**) DF-CS_H_, (**B**) DF-CS_M_, (**C**) DF-CS_L_ and (**D**) DF-CMCS. In all cases: Pure diflunisal (▲), kneaded (○), physical mixture (♦) and coevaporated systems (□).

DF release from DF-CS and DF-CMCS solid dispersions have been studied with the aim of evaluating the influence of both preparation methods (co-evaporation and kneading), and therefore the drug’s crystalline state on DF release. Furthermore, the dissolution, behaviour was analysed according to the polymer employed. The comparative study was completed with the dissolution analysis of pure diflunisal and the corresponding physical mixtures. The dissolution tests were performed over 12 h. as shown in Figure 1.

It can be observed that the release rate of DF increases with decreasing the molecular weight of chitosan and this consideration is valid for all the samples whatever preparation method is employed. The viscosity of the gel layer increases with the polymer molecular weight, therefore, the dissolution rate of the drug is slowed down. Nevertheless, the highest drug release was detected from systems containing CMCS because of its higher aqueous solubility with respect to chitosan polymer.

On this matter, it should be noted that all DF-CMCS systems present similar release profiles with overlapping traces, and when the dissolution efficiency values at 12 h were compared, the variation between the different preparation methods was less than 2% (KN = 78.9%, PM = 76.9%, CO = 78.2%), which clearly indicates the similarity among these profiles. In light of these results, it can be concluded that the method of preparation of the solid dispersions, for the conditions evaluated as part of this work, does not influence the release profile of diflunisal from the DF-CMCS systems, including drug-polymer physical mixtures.

However, when the focus was placed on the influence of the drug crystallinity associated to the solid dispersion preparation method for chitosan samples, the highest diflunisal delivery rate was obtained from co-evaporated systems (crystalline), followed by physical mixtures and kneaded products (amorphous). This trend is observed for the three chitosans tested but not for the DF-CMCS systems, as previously mentioned.

The dissolution efficiencies values differed by more than 28% in all the pairwise comparisons, except when DF-CS_L_ PM and CO were compared (7.6%).

Table 1 shows the comparison parameters for DF release profiles from the DF-CS systems by pairwise procedures. Significant differences between the co-evaporated, physical mixture and kneaded dissolution profiles can be appreciated from the Rescigno index (*ξ*) and the difference factor (*f*_1_) data. The only exception is the similarity obtained between the physical mixture and the co-evaporated system with low molecular eight chitosan (*f*_1_ value lower than 15 and *ξ* close to 0). The lower viscosity of this polymer, and thus the higher dissolution rate for both samples could be the main reason for this similar dissolution behaviour

The results obtained from *f_1_* and Rescigno index (*ξ*) (Table 1) are in accordance with those obtained from the dissolution efficiencies.

As can be observed in Figure 1, the preparation method and thus, drug crystallinity lead to different release profiles; therefore, it is necessary to delve into the causes that produce the differences among these drug delivery systems.

In accordance with the crystalline state of drug molecules in DF-CS solid dispersions, it could be expected that DF release rate would be faster for kneaded products due to their amorphous state. In addition, it could be predicted that coevaporated systems, which showed a uniform dispersion of DF crystals on the polymer surface, could show a higher dissolution rate than that for PM, characterized by large aggregates of drug crystals, as observed by SEM micrographs reported in a previous study [15].

However, as has been mentioned, the highest DF release rate was found for DF-CS CO, followed by PM and KN. This trend was observed for the three different chitosan molecular weights tested. As the preparation method performs an important role on DF release rate that cannot be explained merely through the drug crystalline state obtained as result of this process, it is necessary to delve into the causes that lead to unexpected drug delivery profiles.

An explanation for the obtained results could be associated with the fact that the solid dispersion preparation conditions would lead to different microstructures, which might determine the accessibility of the solvent to drug molecules, leading to significant changes in DF release rate. Therefore, the next step in our study was to analyse the pore structure or microstructure of these systems.

### 2.2. Influence of Microstructure on Drug Release

The internal structures of the compressed samples have been studied in order to establish a possible relationship between the structural characteristics of dispersions and the unexpected slow-release behaviour of the KN systems with respect to the PM and CO solid dispersions. The microstructure of these products was analysed by mercury intrusion porosimetry. This is a void-space sensitive technique that provides an accurate measure of the pore size distribution and pore volume (porosity), supplying information about the water diffusivity through the porous matrix [17]. In this regard, a higher empty volume would make easier the solvent access and thus, the drug diffusion through larger pores. In addition, pore size distribution data can be obtained due to the pressure required to intrude mercury into sample, being inversely related to the sample pore size [18].

**Figure 2:**
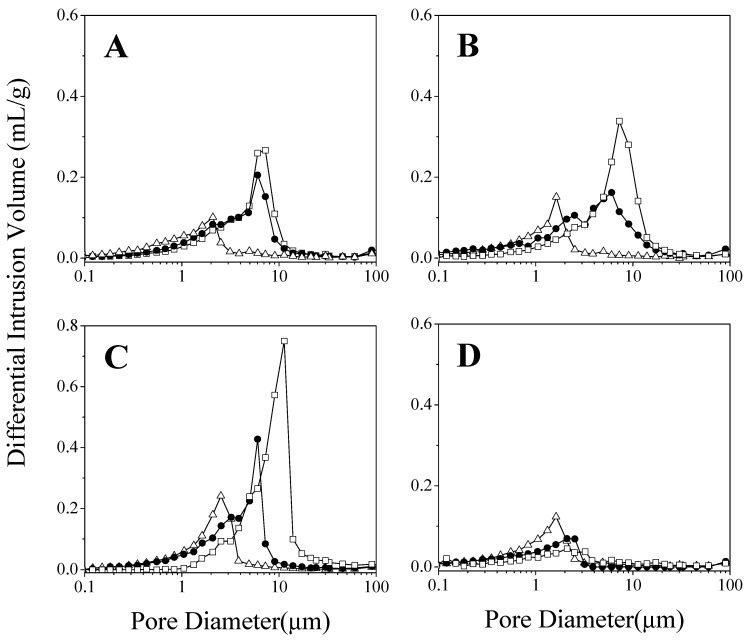
Pore mean diameter distributions of (**A**) DF-CS_H_, (**B**) DF-CS_M_, (**C**) DF-CS_L_ and (**D**) DF-CMCS. In all cases: KN (∆), PM (●) and CO systems (□).

Figure 2 shows the mean pore size distributions of the samples, which provides pore diameters and pore abundance associated to peak height. Sharp unimodal pore size distribution patterns were found for all the samples analysed. DF-CS KN presented the lowest mean pore size (1–3 μm) together with a low void volume, whereas DF-CS CO exhibited the opposite trend, a high number of large size pores (8–10 μm) that corresponded to a greater empty volume within the disc. Moreover, the corresponding PM showed intermediate mean pore size diameters (5–8 μm) and empty volumes. These considerations can be applied to the three different chitosan molecular weights employed in this work. However, it is noteworthy that all DF-CMCS systems showed similar pore size distributions with small pores (1–2 μm) and low empty volume. This fact could be associated with the higher solubility of CMCS in comparison with CS, which would imply that the preparation method does not lead to differences in the porous microstructure of these systems.

It should also be noted that the parameters that complete the internal structure characterization of the samples are the total intrusion volume, porosity, tortuosity factor, and permeability (Table 2). As previously explained, all these structural factors could significantly determine the drug dissolution processes (according to Equation (13)—Material and Methods) and thus, could explain the drug release performance.

The total intrusion volume values of DF-CS-CO, as well as their mean pore size distributions, reveal that the solvent evaporation process leads to a larger empty volume than that found in the kneaded samples. In addition, the low tortuosity factor indicates that the dissolution medium can be incorporated into the matrix in a more straightforward way, and, therefore, faster in co-evaporated products. The experimental data obtained on the porous structure of the kneaded solid dispersions showed low intrusion volumes and permeability values together with a high tortuosity, which resulted in a more difficult access of the solvent to the drug. The physical friction treatment during the kneading process could be the main cause of porosity reduction, in contrast with the solvent evaporation in CO systems. Finally, it should be noted that the pore microstructural parameters of the physical mixture presented intermediate values for each parameter and, therefore, an intermediate drug release profile. These considerations are valid for the three molecular weights of chitosan.

From the pore microstructural analysis, it can be concluded that the porous structure plays an important role in the dissolution rate of diflunisal and may well explain the release behaviour of the different systems. Summarizing, it is evident that the solid dispersion preparation method conditions the porous structure and, therefore, the release rate of diflunisal. The co-evaporation process involves a fast solvent elimination, which causes an increase in the porosity of the systems and the possible formation of channels in the solid dispersion, in contrast with the kneading treatment.

Concerning the influence of the polymer chains’ length, the higher the molecular weight, the lower the porosity and permeability and the higher the tortuosity factors (Table 2). Once again, these considerations are valid for all the different chitosan solid dispersion, regardless of the preparation method employed.

**Figure 3:**
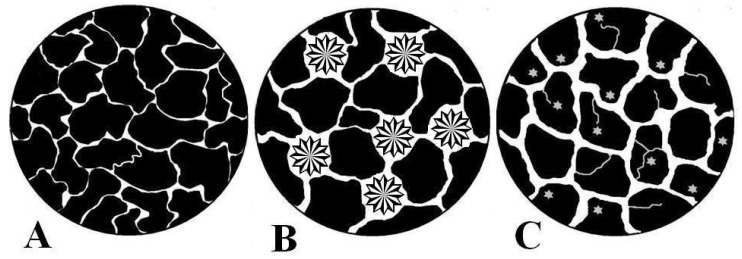
Schematic representation of the microstructure for DF-CS discs prepared by KN (**A**), PM (**B**) and CO (**C**). DF form II agglomerates (🟔). DF form III dispersed crystals (🟌).

The internal microstructure strongly influences the access of the solvent and the subsequent drug release to the medium through the channel-type structure [19]. Figure 3 shows a schematic representation of the possible structure of the systems tested. This representation takes into account the information obtained about the microstructure analysed in the current investigation together with the SEM images shown in our prior paper [15].

Finally, all the DF-CMS systems exhibited similar characteristics: slightly porous structure and small mean pore size (1–2.5 μm). The overlapped drug release profiles can be associated with this fact, irrespective of the different crystalline structures.

It is noticeable that with similar or even smaller values of porosity than kneaded DF-CS systems, the dissolution profiles of DF-CMCS are very different from those of CS systems. This behaviour can be attributed to the higher solubility of the carboxymethylated derivative in aqueous media. Therefore, it is likely that the dissolution of the drug would take place by a different mechanism, as will be analysed and discussed in the following section.

### 2.3. Mathematical Models

DF release profiles of DF-CS and DF-CMCS samples were first fitted to the Higuchi equation as well as to first and zero order kinetic models. The defined geometry of the samples allowed the use of mathematical models to obtain information concerning the processes involved in drug release, such as diffusion and/or erosion/relaxation [20]. It should be noted that none of the dissolution profiles exhibited first order release kinetics, and some of them were properly adjusted to the Higuchi equation (R^2^ > 0.99), which means a prevalence of diffusion processes on drug release. Nevertheless, DF-CMCS systems showed near zero order release kinetics (R^2^ > 0.98).

Furthermore, Korsmeyer-Peppas and Peppas-Sahlin equations were used to delve into the mechanisms involved in the DF release from CS and CMCS polymeric systems (Table 3). As can be seen, all the dissolution profiles showed a good fit to both equations (R^2^ = 0.98–0.99). The DF-CS_L_ *n*-values calculated from the Korsmeyer-Peppas equation approached the hypothetical pure diffusion exponent for our tablets (*n* = 0.475), indicating that diffusion was the main driving force in the release process. On the other hand, DF-CS_M_ and DF-CS_H_ showed *n*-values slightly higher than those found for DF-CS_L_, which would be indicative of a small contribution of the polymer erosion, probably associated with a more difficult access of solvent as a result of the microstructure.

In addition, the compact structure of the polymer chains in DF-CS_H_ samples would make difficult the release of drug molecules from the matrix through a pure Fickian diffusional process. On the contrary, semi-flexible linear chains of chitosan at low molecular weight (DF-CS_L_ products) would allow an easier diffusion of DF molecules to the dissolution medium [21].

This trend was observed for all DF-CS systems regardless of the preparation method employed. In order to quantify the relative contributions of diffusion and erosion for each system, the dissolution data were fitted to the Peppas-Sahlin equation (Equation (8)) (Table 3).

It was found that the diffusion constant values (*k_D_*) were markedly higher than the erosion constants (*k_E_*) in the cases of all DF-CS systems. The small contribution of the erosion process agrees with the slightly higher *n*-values with respect to 0.475, *n*-reference value for pure Fickian diffusional process in our discs. A markedly different behavior was observed in the release profiles for DF-CMCS samples in comparison with DF-CS discs. As mentioned above, DF-CMCS release profiles nearly fit to the zero-order kinetic model (R^2^ = 0.98–0.99), which might agree with the diffusion and erosion contributions calculated from the Peppas-Sahlin model. The *n*-values above 1.0 in these systems are ascribed to the so-called Super-case II transport, which suggests that DF was mainly released by relaxation/erosion of the polymer chains. It is supported by the larger water solubility achieved by the carboxymethylated derivative with respect to the unmodified chitosan [22]. The high *k_E_*-values (*k_E_* = 22.6–24.5) would indicate that the erosion of CMCS plays a major role in diflunisal release from these systems, whereas the negative values obtained for *k_D_* can be attributed to a negligible contribution of drug diffusion compared to the relaxation mechanism from the swollen polymer [23].

**Figure 4:**
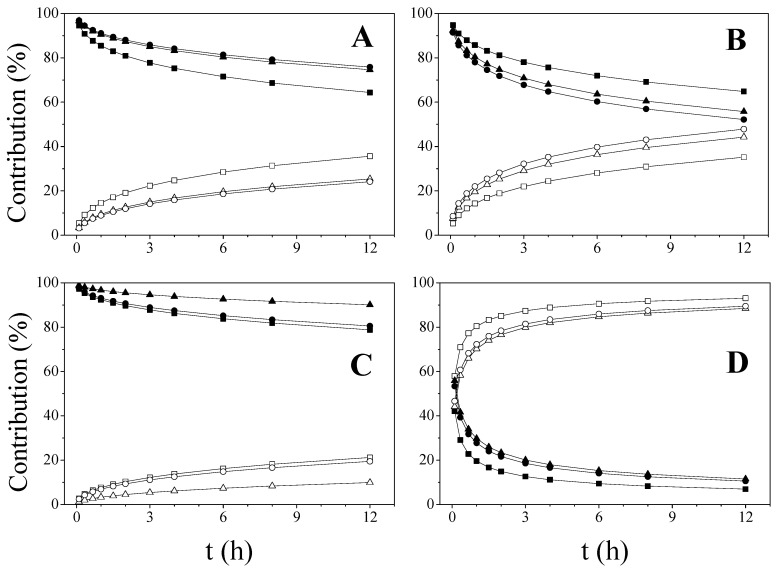
Contribution of the diffusion and erosion mechanisms for DF release from: DF-CS_H_ (**A**), DF-CS_M_ (**B**), DF-CS_L_ (**C**) and DF-CMCS (**D**). In all cases: diffusion contribution: KN (▲), PM (●) and CO discs (■) and erosion contribution: KN (∆), PM (○) and CO discs (□).

The diffusion (*D*) and erosion (*E*) relative contributions (Equation (9a,b)—Material and Methods) were calculated from the fitting of kinetic data (*K_D_* and *K_E_*) (Figure 4).

As it has been mentioned, the diffusion mechanism is predominant during the entire dissolution process in DF-CS systems. In addition, the process of erosion/relaxation becomes more important for the matrices prepared with CS_M_ and CS_H_ as drug release progresses. This fact could be attributed to the higher viscosity of the layer formed by the polymer in contact with the dissolution medium. As expected, the erosion contribution in DF-CMCS predominates over diffusion throughout the entire dissolution tests, when the drug is either mixed or dispersed in the soluble chitosan derivative.

## 3. Materials and Methods

### 3.1. Materials

Diflunisal (DF) polymorph II was kindly supplied by Merck Sharp and Dohme (Madrid, Spain). Chitosan of low (CS_L_) (50–190 kDa), medium (CS_M_) (190–310 kDa) and high molecular weight (CS_H_) (>375 kDa) was supplied by Aldrich (Madrid, Spain). The degree of deacetylation for the three polymers was 75–85%. O-Carboxymethylchitosan (CMCS) was supplied by Heppe Medical Chitosan GMBH (Halle, Germany). This polymer presented 80–95% degree of deacetylation and a molecular weight of 51 kDa. The reagents ethanol (Scharlau, Barcelona, Spain) and hydrochloric acid (Panreac, Barcelona, Spain) were used as received. Aqueous solutions were prepared with deionised water obtained from a commercial Millipore Elix 3 system (0.1 μS/cm conductivity). All reagents and chemicals used were of analytical grade.

### 3.2. Preparation of Solid Dispersions

Diflunisal-chitosan (DF-CS) and diflunisal-carboxymethylchitosan (DF-CMCS) solid dispersions (30:70 drug/polymer ratio) were prepared using two different methods: kneading (KN) and co-evaporation (CO). The preparation of solid dispersions was fully described in our previous paper focused on the diflunisal polymorphism and solubility in the presence of chitosan polymers [15]. Briefly, KN products were prepared by wetting a mixture of DF and either CS or CMCS with an ethanol/water solution (50% *v*/*v*) until a dense paste was obtained, whereas the DF-CS CO samples were prepared by adding an ethanolic solution of DF to CS or CMCS dissolved in aqueous solution of hydrochloric acid (pH = 1.2). The solvent removal was carried out at 60 °C in a rotary evaporator (Buchi R-3000, Flawil, Switzerland).

The corresponding physical mixtures (PM) were prepared as well for comparison purposes.

### 3.3. Dissolution Rate Studies

The in vitro dissolution studies of pure DF, drug-polymer solid dispersions, and physical mixtures were performed employing discs as drug delivery devices. Briefly, 200 mg of the samples were directly compressed in a Perkin Elmer (Überlingen, Germany) hydraulic press supplied with a 13 mm diameter punch (compaction pressure 753 MPa) to obtain the compressed samples. The particle size and compaction conditions were maintained constant, as it has been shown that an increase in compression force, dwell time, or strain rate can modify microstructural properties of the systems, such as porosity and pore diameter [24].

The dissolution rate assays were carried out from discs according to the USP43-NF38 (2020) paddle method using a Sotax AT7 smart Dissolution Testing Unit (Sotax, Bern, Switzerland) provided with a Sotax fraction collector and online filtration (membrane filter 2.0 μm). The dissolution conditions were: 900 mL of deionized water as dissolution medium, 37.0 ± 0.2 °C and 50 rpm. The amount of drug released was determined spectrophotometrically (Hewlett Packard 8452 diode-array spectrophotometer, Madrid, Spain) at 252 nm. Each dissolution test was performed at least in triplicate (CV < 5%).

#### 3.3.1. Release Parameters

Kinetic parameters such as dissolution efficiency, difference factor (*f*_1_), and the Rescigno index (*ξ*) were analyzed in order to compare the release profiles of all the systems tested.

Dissolution efficiency (*DE*) is defined as the area under the dissolution curve up a certain fixed time, and it is expressed as a percentage of the area of the rectangle described by 100% dissolution at the same time [25]:

(1) $$DE=\frac{\int_{0}^{t}y\times dt}{y_{100}\times t}\times 100$$

The difference factor (*f*_1_) is a simple pairwise procedure that measures the average percentage difference over all time points between two dissolution curves. It can be calculated using the difference between each individual point of the kinetic profiles or between the areas under the curves [26]. This factor is recommended by the FDA [27] to compare two dissolution profiles, being necessary to consider one of them as a reference product:

(2) $$f_{1}=\frac{\sum_{j=1}^{n}\left|R_{j}-\left.T_{j}\right|\right.}{\sum_{j=1}^{n}R_{j}}\times 100$$

where *n* is the number of sampling points, *R_j_* and *T_j_* are the percent dissolved of the reference and test products at each time point *j. f*_1_ values lower than 15 indicate similar profiles.

The Rescigno index (*ξ*) was proposed as a bioequivalence index, which can also be used to compare the release profiles of drugs [28]:

(3) $$\xi_{i}={\left\{\frac{\int_{0}^{\infty }{\left|M_{R}\left(t\right)-M_{T}\left(t\right)\right|}^{i}dt}{\int_{0}^{\infty }{\left|M_{R}\left(t\right)+M_{T}\left(t\right)\right|}^{i}dt}\right\}}^{1/i}$$

*M_R_*(*t*) and *M_T_*(*t*) being the amount of drug release at each sample time for reference and test product, respectively. The Rescigno index is 0 when the two release profiles are identical. The integer number *i* is fixed at 1.0 for drug release profiles. The Rescigno parameter was calculated from the sum of areas in the (*M*, *t*) plane enclosed by the two curves being compared. All the areas are considered positive.

Origin Pro 8.5 software was employed for the calculations of area under the curves (DE, *f*_1_ and Rescigno).

#### 3.3.2. Mathematical Models

The experimental data were fitted to different kinetic equations in order to obtain the mathematical model that best describes the drug release process. In addition, some considerations about the mechanisms involved in drug release were discussed.

The model dependent methods applied to this study were zero and first order kinetics together with Korsmeyer-Peppas, Higuchi, and Peppas-Sahlin equations. For all the kinetic models tested, a direct non-linear fitting of the experimental data was carried out in which only the points with *M_t_/M*_∞_ < 0.6 were used. The zero-order kinetic model is used for systems where matrix releases slowly the same amount of drug per unit of time [29].

(4) $$\frac{M_{t}}{M_{\infty }}=k_{0}t^{}$$

and the first-order is described by the following equation:

(5) $$\ln\left(\frac{M_{t}}{M_{\infty }}\right)=k_{1}t^{}$$

where *M_t_/M*_∞_ is the drug release fraction at time *t*, *k*_0_ the zero-order and *k*_1_ the first-order release constants, respectively.

The Higuchi model describes kinetics in which the drug is released by pure Fickian diffusion mechanism, *k_H_* being the diffusion constant [30]:

(6) $$\frac{M_{t}}{M_{\infty }}=k_{H}t^{1/2}$$

The Korsmeyer-Peppas equation is a simple semi-empirical model, which relates the amount of drug released with the elapsed time [31]:

(7) $$\frac{M_{t}}{M_{\infty }}=k_{KP}t^{n}$$

where *k_KP_* is a constant incorporating the structural and geometrical characteristics of the matrix system and *n* is the release exponent, which is associated with the drug release mechanism and relies on the geometry of the device. The drug release is diffusion controlled if the n value is close to 0.5 (the exact value depends on geometry), and anomalous transport (non-Fickian) is observed for an n value between 0.5 and 0.89. In our systems, the *n* value considered was 0.475. This value was obtained from the plot of aspect ratio (diameter/thickness) against the diffusional exponent [32].

The analysis of release mechanism was carried out according to Peppas-Sahlin equation, which provides information about the relative contributions of both diffusion and erosion phenomena when the *n* value obtained from kinetic fittings was greater than 0.475. The Peppas-Sahlin model fitted to the experimental data provides values of both diffusion constant (*k_D_*) for the Fickian contribution and erosion constant (*k_E_*) for the erosional/relaxational contribution; the *m* exponent is 0.475 for our systems, as previously mentioned:

(8) $$\frac{M_{t}}{M_{\infty }}=k_{D}t^{m}+k_{E}t^{2m}$$

Erosion (*E*) and diffusion (*D*) contributions can be calculated from the data of the kinetic constants obtained as follows [33]:

(9a) D=11+kEkDtm

(9b) 1=D+E

### 3.4. Mercury Intrusion Porosimetry

Pore size distribution tests of the compressed samples were performed using an AutoPore IV 9500 Mercury Intrusion Porosimeter (MIP) (pressure range between 0.0015 and 207 MPa). Pressure, pore diameter, and intrusion volume were automatically registered.

The pressure required to intrude mercury into de sample’s pores is inversely proportional to the pore sizes (*r*) as described by Washburn’s equation [34]

(10) $$P=-\frac{2\gamma \cos\theta }{r}$$

where *γ* is the surface tension and *θ* is the contact angle. From the data obtained (software treatment), the permeability and tortuosity factors were calculated as well.

Initially, Noyes and Whitney described a model in which the mass transport by diffusion was postulated as the rate-limiting step controlling the dissolution rate of a substance.

(11) $$\frac{dc}{dt}=K\left(C_{S}-C_{t}\right)$$

where *C_s_* is the drug solubility, *C_t_* is the concentration at time *t*, and *K* is a constant.

Subsequent interpretations of this equation gave rise to more specific models that incorporated different processes involved in drug release. In this sense, the Higuchi equation was proposed for systems in which release occurs exclusively on the surface of the system, with a content of drug in the matrix above the solubility and assuming pseudo-steady state [35].

(12) $$\frac{M_{t}}{A}=\sqrt{D\left(2C_{0}-C_{S}\right)C_{S}t}$$

where *M_t_*/*A* is the cumulative amount of drug released per unit of area, *A* is the surface, *D* the diffusion coefficient and *C_0_* the initial concentration of the drug.

However, for more complex systems where diffusion occurs through porous matrices, so factors such as tortuosity, permeability or porosity should be considered in order to obtain a better approach to the real delivery process [36]. In this sense, considering the water penetration and drug diffusion out of the porous structure as important factors, the following equation was proposed [37]:

(13) Mt=Dετ2A−εCsCst12

where *τ* is the tortuosity (Equation (14a)) that is related with the constriction factor (*σ*) (Equation (14b)):

(14a) $$\tau =tortuosity=\frac{dist_{traveled}}{dist_{shortest}}$$

(14b) $$factor=\frac{\tau }{\sigma }$$

and *ε* is the porosity (Equation (15)).

(15) $$\varepsilon =porosity=\frac{Volume_{void}}{Volume_{total}}$$

The diffusion coefficient, which indicates the ability of a material to be traversed by a fluid, is related to the permeability through the solubility coefficient (*S*) [19]:

*P* = *permeability* = *D* × *S* (16)

## 4. Conclusions

The in-depth analysis of diflunisal release has evidenced that the general theory that predicts higher release rates for amorphous solid dispersions in comparison with crystalline products cannot be applied to the diflunisal-chitosan systems studied. The knowledge of microstructure is of paramount importance to analyze the anomalous or non-expected results in the dissolution behavior of diflunisal from chitosan solid dispersions, when they cannot be explained in terms of crystallinity. Mercury intrusion porosimetry has proven to be an adequate method to obtain information about the microstructure of these systems. The porous structure is conditioned by the solid dispersion preparation method and plays an important role in the drug dissolution rate that explains the release mechanism.

The higher porosity and lower tortuosity obtained for DF-CS co-evaporated solid dispersions in contrast to DF-CS kneaded products, leads to a faster solvent penetration into the matrix and provides a higher dissolution rate. This behavior was similar for all chitosan systems regardless of the molecular weight of the polymer. However, the high aqueous solubility of carboxymethylchitosan gives rise to similar microstructures and dissolution profiles irrespective of the preparation method. The mechanism of difunisal release depends on the polymer employed. It is mainly diffusion controlled by chitosan solid dispersions, whereas the erosion/relaxation of the polymer plays a major role in carboxymethylchitosan systems.

## Figures and Tables

**Table 1:** Comparative parameters for diflunisal release profiles.

Tablet	Profiles Compared	*f*_1_	Rescigno Index (*ξ*)
DF-CS_H_	KN vs. PM	40.22	0.24
	PM vs. CO	23.45	0.13
DF-CS_M_	KN vs. PM	56.69	0.35
	PM vs. CO	27.31	0.16
DF-CS_L_	KN vs. PM	51.64	0.34
	PM vs. CO	10.03	0.09

**Table 2:** Internal structure parameters of DF-CS samples.

Sample		Total Intrusion Volume	Porosity	Permeability	Tortuosity Factor
		(mL/g)	(%)	(MDarcy)	
DF-CS_H_	KN	0.083	09.12	0.44	2.17
DF-CS_H_	PM	0.097	13.10	7.98	2.16
DF-CS_H_	CO	0.109	13.59	17.07	2.11
DF-CS_M_	KN	0.090	12.62	0.61	2.16
DF-CS_M_	PM	0.115	15.05	7.00	2.14
DF-CS_M_	CO	0.187	20.28	27.89	2.09
DF-CS_L_	KN	0.119	15.08	1.87	2.14
DF-CS_L_	PM	0.172	19.78	13.79	2.10
DF-CS_L_	CO	0.291	30.51	87.22	2.01
DF-CMCS	KN	0.083	10.37	0.49	2.21
DF-CMCS	PM	0.067	08.81	0.54	2.15
DF-CMCS	CO	0.057	07.83	0.52	2.18

**Table 3:** Fitting results of the experimental diflunisal release data to Korsmeyer-Peppas and Peppas-Sahlin equations. Data represent the mean of at least 3 experiments.

Tablets		Korsmeyer-Peppas			Peppas-Sahlin (m = 0.475)		
		*n*	*k_KP_* × 10^2^ (h^−n^)	R^2^	*k_D_* × 10^2^ (h^−m^)	*k_E_* × 10^2^ (h^−2m^)	R^2^
DF-CS_H_	KN	0.58 (±0.02)	17.0 (±0.6)	0.994	14.7 (±1.0)	2.5 (±0.5)	0.994
	PM	0.54 (±0.01)	23.2 (±0.3)	0.999	21.1 (±0.7)	2.2 (±0.4)	0.998
	CO	0.54 (±0.02)	23.6 (±0.5)	0.996	21.5 (±1.1)	2.1 (±0.6)	0.996
DF-CS_M_	KN	0.58 (±0.02)	17.4 (±1.2)	0.996	15.0 (±0.8)	2.5 (±0.4)	0.996
	PM	0.58 (±0.05)	40.0 (±1.2)	0.987	32.0 (±5.6)	7.8 (±4.8)	0.985
	CO	0.59 (±0.05)	40.7 (±0.8)	0.989	31.6 (±5.4)	8.9 (±4.6)	0.986
DF-CS_L_	KN	0.51 (±0.03)	51.2 (±0.3)	0.997	47.2 (±4.2)	3.9 (±4.0)	0.996
	PM	0.49 (±0.01)	64.2 (±0.9)	0.999	62.1 (±1.4)	2.1 (±1.6)	0.999
	CO	0.44 (±0.04)	41.0 (±0.6)	0.991	44.5 (±3.8)	3.3 (±3.2)	0.992
DF-CMCS	KN	1.06 (±0.03)	17.0 (±1.1)	0.998	-	22.6 (±1.1)	0.997
	PM	1.17 (±0.10)	14.1 (±1.2)	0.977	-	24.3 (±2.8)	0.978
	CO	1.14 (±0.06)	15.2 (±1.1)	0.991	-	24.5 (±1.9)	0.991

(-) negative *k_D_* values.
